# Effects of Age and Playing Tactics on the Individual Tactical Behavior in U10 and U12 Elite Spanish Soccer Players

**DOI:** 10.3390/sports10110185

**Published:** 2022-11-21

**Authors:** Joaquín González-Rodenas, Gonzalo Pedrera, Víctor Dorado, Rodrigo Aranda-Malavés, Andrés Tudela-Desantes, Pedro De Matías-Cid

**Affiliations:** 1 Centre for Sport Studies, Rey Juan Carlos University, 28942 Madrid, Spain; 2 Department of Physical Education and Sports, University of Valencia, 46010 Valencia, Spain

**Keywords:** youth football, match analysis, technical demands, observational methodology, athlete development

## Abstract

The aim of this paper was to explore the effects of age and playing tactics on the individual tactical behavior and performance in young soccer players. A total of 1247 individual possessions during 16 knockout matches from *LaLiga Promises U12 tournament* (n = 621) and *LaLiga U10 Iscar Cup* (n = 626) were analyzed by observational methodology. Multilevel and multivariate logistic regression models were created to explain the interdependent effects of age category and playing tactics on the individual tactical behavior and performance. Youth players performed most of their actions against defensive pressure (72.5%), during offensive support (91.3%) and receiving the ball facing forward (62.6%). The most frequent action was to receive and pass the ball (69.6%) and the level of offensive success was 56.9%. The multilevel mixed models revealed that U10 players presented higher odds of running with the ball vs. passing the ball (OR = 1.823; 95% CI: 1.333–2.493; *p* < 0.001) and lower odds of achieving offensive success (OR = 0.698; 95% CI: 0.525–0.928; *p* < 0.05) than U12 players. Regarding playing tactics, technical-tactical dimensions such as the players’ body shape when receiving the ball, offensive support, defensive pressure, collective type of attack and type of technical action presented a significant and combined effect on the offensive success regardless of age category, playing position and match status. This study found tactical differences between U10 and U12 age categories and revealed important interactive effects of multiple tactical dimensions on the individual offensive behavior and performance in youth soccer players.

## 1. Introduction

Technical and tactical analysis in professional soccer has increasingly grown in the last decade [1]. In this sense, the proliferation of technological systems such as global positioning systems (GPS), Prozone_STATS, OPTA, etc. that collect performance data has led to an exponential development and sophistication of data analysis techniques [2]. This progress in technical and tactical knowledge aims to improve the training design and match strategy, contributing to a more specific technical and tactical development of professional players.

However, this exponential progress in professional soccer contrasts with a limited amount of research on technical and tactical analysis in youth soccer players [3], and especially in prepubescent players. In this sense, the existing studies have mainly focused on physiological and physical variables, while the technical-tactical insights during competition are still scarce [4,5,6]. This fact could be partly due to the lack of technological systems and data analysts in youth teams, which makes it very difficult to collect and analyze data from training sessions and matches. In this context, most of the individual skills assessments in youth soccer seem to be performed in settings that are unrepresentative of soccer match play [7]. In fact, the review of Aquino et al. [8] observed that almost 90% of studies that evaluated skill-related performance were carried out outside of the real-match context. For this reason, the existing literature about technical and tactical analysis in youth soccer has required the creation of specific observational tools to evaluate individual and collective tactical behaviors [9].

From this perspective, recent studies have focused on the implementation of modified but representative versions of soccer such as small-sided games to evaluate the individual performance of young soccer players [10]. For instance, da Costa, Garganta, Greco, Mesquita & Alfonso [11] observed that as the age group increased from U11 to U20, the players had greater participation in the game, performed more tactical actions related to the width and length and defensive unity. This study also found that there were less inter-individual variances in the older groups and overall better performance, probably reflecting the learning progress of the players. Regarding the very young ages, existing studies have revealed that prepubescent players (U8–U10) display a more vertical, offensive and individualistic (dribbling) behavior [12]. In this sense, at the age of U12, a boost of tactical performance seems to emerge so that players are able to play a more combinative play with more collective width and better efficacy in the use of different technical-tactical skills [13]. In this way, it seems that between U10 and U12 age groups, there exists an important evolution in the technical and tactical performance of players, which probably reflects the progression in learning and understanding of the principles of the game.

However, despite the important information and application provided by previous studies focused on small-sided games, the research on tactical analysis in youth soccer is still very limited in comparison to professional soccer [8]. Furthermore, it is surprising the existence of very few studies that evaluate the technical and tactical demands of youth players in the actual competition, which allows researchers to analyze the players under real contextual physical, spatial and time constraints.

Therefore, the aim of this paper was to explore the effects of age, playing position and playing tactics on the individual tactical behavior and performance in U10 and U12 elite soccer players in real competition. Our hypothesis contends that U10 and U12 age-groups present differences regarding the technical-tactical actions and their offensive success. Moreover, we hypothesize that the tactical performance and offensive success of youth soccer players are explained by the interactive effects of spatial, collective, individual and defensive constraints.

## 2. Methods

### 2.1. Sample

A total of 16 knockout matches from the official tournaments of the Spanish *LaLiga* in youth soccer were evaluated (8 matches: round of 16 of *LaLiga Promises U12 soccer-7 tournament*; 8 matches: round of 16 of *LaLiga Iscar Cup U10 soccer-7 tournament*) where a total of 32 teams and 320 players (excluding goalkeepers) participated during the games. The duration of the matches was 24 min with 2 halves of 12 min each. The videotapes of the matches were obtained from a live TV broadcast. Ethical approval was not required for this study because the tactical analyses of players were performed in matches that were recorded from TV broadcasters and the videos were public.

The unit of analysis was the individual ball possession (IBP), described by Link and Hoernig [14] as the time that begins the moment a player is able to perform an action with the ball (following an IBP of another player or a game interruption), and it ends the moment IBP for another player begins. To obtain a representative sample of IBPs of each match, 5 consecutive minutes of each half were selected randomly, and the totality of IBPs performed during these periods were evaluated.

Finally, a total sample of 1247 IBPS were analyzed (U12 = 621, U10 = 626).

### 2.2. Dimensions

A total of seven technical-tactical dimensions related to the beginning, development and the end of the IBP were examined (Table 1). These dimensions were selected from the INDISOC observational tool [15] that provides a theoretical framework to evaluate the individual behavior in competitive soccer by analyzing the interdependency of individual, environmental and task constraints, as previous studies claimed [16,17].

### 2.3. Match Performance Analysis

The study was based on observational methodology [18]. The observation design is punctual, because the data collection takes place in one single session, idiographic, because it is focused on a study unit (the player) and multidimensional, because the player’s performance is based on various criteria [19].

For the analysis, two observers were trained in the use of the INDISOC tool for four weeks by the principal researcher of this study, who holds wide experience in performance analysis in soccer and is one of the lead authors of the used tool. This training included theoretical and practical lessons. When the training was completed, each observer analyzed 8 matches separately. The analysis was made post-event and each IBP was analyzed as many times as necessary for the observers. The Lince-Plus software [20] was used to register and code the data.

As for the reliability of the data, the two observers in addition to the principal researcher (used as reference) analyzed 163 IBPs which corresponded with two matches for the analysis of inter-observer reliability. Subsequently, the principal researcher re-observed the game three weeks later for the intra-observer concordance. Kappa correlation coefficients (κ) were calculated for inter-observer and intra-observer reliability. In this sense, this analysis showed an appropriate level of reliability according to Altman criteria [21] (inter-observer Kappa coefficient = 0.81–0.97; intra-observer Kappa coefficient = 0.85–0.99).

### 2.4. Statistical Analysis

The SPSS software was used to perform the analyses (IBM SPSS, Version 20.0). Firstly, an analysis of frequencies was carried out to describe the characteristics of the sample and the occurrence of each tactical dimension according to the age category and playing position.

Secondly, due to the hierarchal structure of the IBPs in soccer (each player plays for a team that has its own tactical style of play), multilevel modeling [25] was carried out to cluster the IBPs performed by players (Level 2) within teams (Level 1). In this manner, a mixed model was created to analyze the effect of the age category and playing tactics (independent variables: fixed effects) on the individual tactical behavior and offensive success (dependent variables), considering the effect of the team identity (random effects). With this organization of the data, binary logistic regressions were constructed to predict the outcome related to the individual tactical behavior (0 = receiving and passing, 1 = running with the ball) and the offensive success (0 = negative outcome, 1 = positive outcome).

For the analysis, unadjusted models (univariate analysis) were carried out to determine the association of each independent variable with the dependent variable. Moreover, adjusted logistic multilevel models (multivariate analysis) were constructed to explore the interdependent effect of the independent variables on the dependent variable. The significance level was set to *p* < 0.050.

Finally, graphic charts with the predicted means were displayed for the variables that presented significant effects on the dependent variables.

## 3. Results

### 3.1. Descriptive Analysis

Table 2 shows the descriptive characteristics of the sample considering the age category and playing position. It can be observed that youth players performed most of their actions against defensive pressure (72.5%), having offensive support (91.3%) and receiving the ball facing forward (62.6%). The most frequent action was to receive and pass the ball (69.6%) and the level of offensive success was 56.9%. U12 players performed more actions in the offensive half (49.6 vs. 41.2%), received the ball less frequently facing backwards (15.0 vs. 20.9%), had less defensive pressure (61.8 vs. 83.1%) and performed more actions related to receiving and passing (73.6 vs. 65.7%), in comparison with U10 players.

### 3.2. Multilevel Regression Analysis

Regarding the random effects, Table 3 shows that the effect of ‘team identity’ did not present a significant variance for the type of action and tactical performance (*p* = 0.481 and 0.542, respectively).

In Table 4, the univariate and multivariate effects of the age category, playing position and several tactical dimensions on the type of action implemented by players can be found. Both age and playing position showed a significant univariate and multivariate effect on the type of action. In this sense, U10 players presented higher odds of running with the ball (OR = 1.823; 95% CI: 1.333–2.493; *p* < 0.001) than U12 players. Furthermore, forward, midfielders and wingers had higher odds of running with the ball, rather than receiving and passing, in comparison with central defenders.

On the other hand, tactical dimensions such as the initial zone, defensive pressure and type of attack presented significant univariate effects on the type of action, but this effect was not significant when the multivariate analysis was undertaken.

The dimension “body shape” did not present a significant effect in the univariate analysis, but its effect was significant in the multivariant model. In this sense, when players received the ball facing forward increased the odds of running with the ball (OR = 1.794; 95% CI: 1.226–2.625; *p* < 0.001), in comparison with receiving the ball facing backwards.

Finally, the dimension “offensive support” shows a significant univariate and multivariate effect on the type of action, so that having offensive support decreased the odds of running with the ball (OR = 0.446; 95% CI: 0.283–0.703; *p* < 0.005) in comparison with not having offensive support.

**Figure 1:**
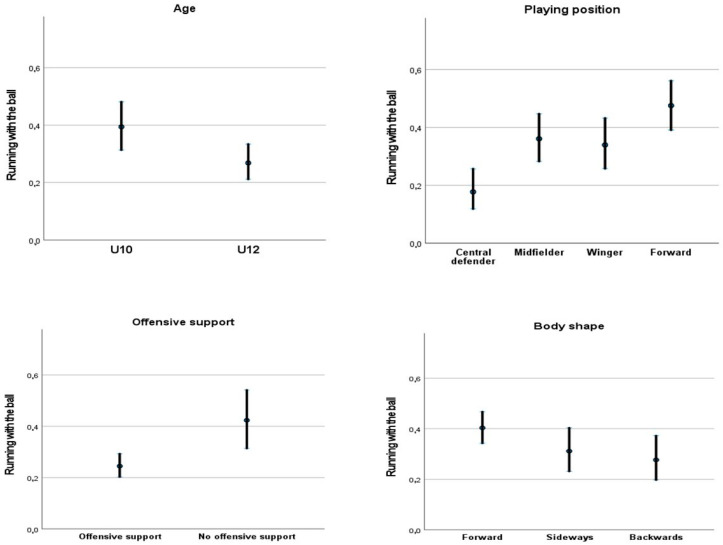
Predicted means and confidence intervals related to the type of action according to different contextual and tactical dimensions after adjusting for the dimensions included in the multivariate analysis.

Following the multilevel and multivariate analysis, Figure 1 displays the predicted means and confidence intervals of the contextual and tactical dimensions that presented significant effects on the type of action. In this sense, U10 players and forwards had the highest predicted levels of actions related to running with the ball, in comparison with U12 players and the rest of the playing positions, respectively. Other categories such as receiving the ball facing forward and not having offensive support presented higher means in relation to the possibility of running with the ball rather than receiving and passing.

Table 5 presents the multilevel model to predict the tactical performance according to the selected contextual and tactical dimensions. Regarding the age effect, U10 players had lower odds of achieving a positive outcome in their actions (OR = 0.698; 95% CI: 0.525–0.928; *p* < 0.05) only when the effect of the rest of the dimensions were considered (multivariate analysis). As for the effect of the playing position, there was a univariate effect that indicated that forwards and wingers had lower odds of having a positive outcome, although the significance of this effect disappeared in the multivariate analysis.

The rest of the tactical dimensions presented significant effects on tactical performance both in the univariate and multivariate analysis. In this sense, playing in the offensive half, receiving the ball facing forward, attacking by counterattack and running with the ball decreased the odds of achieving a positive outcome in comparison with playing in the defensive half, receiving the ball facing backwards, attacking by organized attack and receiving and passing the ball, respectively.

Finally, having offensive support and not having defensive pressure increased the odds of culminating the IBP with a positive outcome.

**Figure 2:**
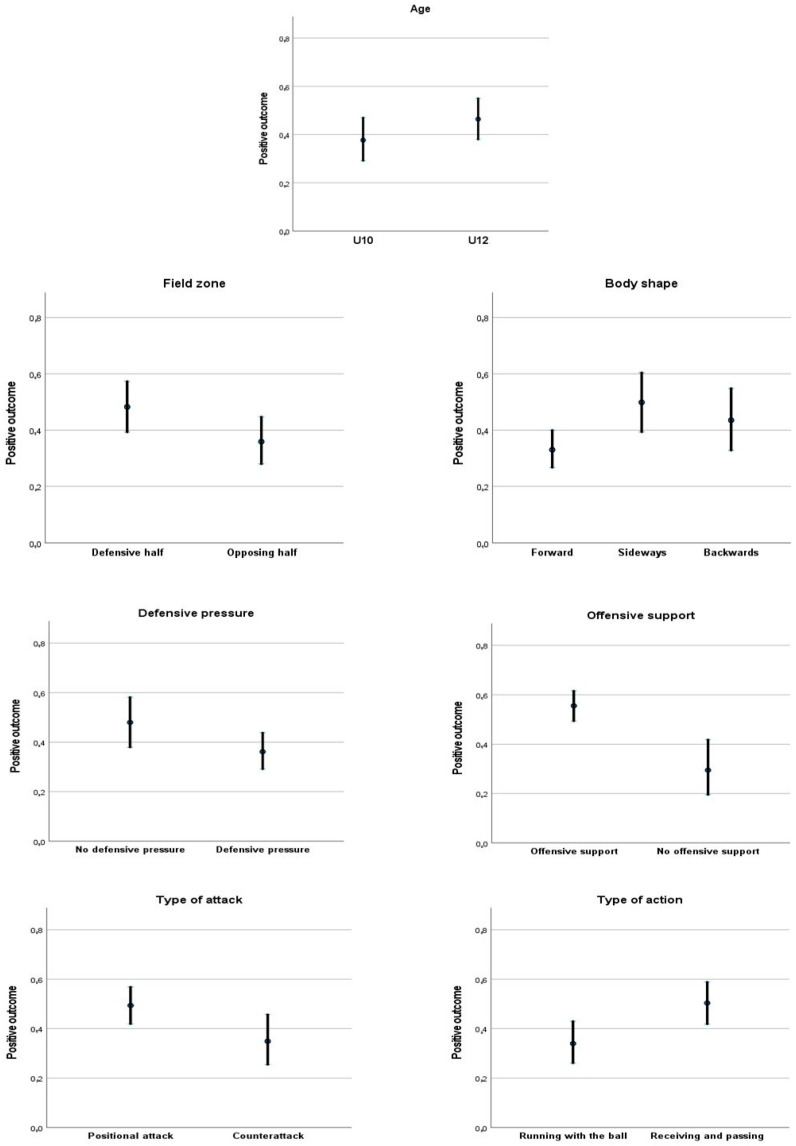
Predicted means and confidence intervals related to the tactical performance according to different contextual and tactical dimensions after adjusting for the dimensions included in the multivariate analysis.

Figure 2 illustrates the predicted means and confidence intervals of the contextual and tactical dimensions that presented significant effects on the tactical performance. It can be observed that categories such as U12 teams, playing at the defensive half, receiving sideways, not having defensive pressure, having offensive support, attacking by organized attack, and receiving and passing the ball register the highest odds of achieving a positive outcome in the IBP.

## 4. Discussion

The aim of this paper was to explore the interactive effects of age, playing position and tactical dimensions on the individual tactical behavior and performance in U10 and U12 elite soccer players in the real competition. Our findings revealed tactical differences between U10 and U12 age-groups, and also observed the significant interactive effects of multiple tactical dimensions including spatial, offensive and defensive constraints on the players’ performance and offensive success.

Regarding the type of action, our data showed that U10 players presented higher odds of running and dribbling with the ball, in comparison with U12 players, regardless of contextual variables, playing position and other tactical dimensions. This finding agrees with previous studies that highlighted that the youngest categories such as U8 and U10 tend to play in a more vertical way, with more individual actions [12,26]. This fact may be associated with the inherent developmental stages of U10 players, which show a more egocentric personality and behavior than U12 players, who are able to collaborate more with their teammates and implement a more collective style of play [13,27].

In addition to the age, the playing position was also a key dimension to understand the type of action implemented by players. In this sense, the multilevel analysis found that midfielders, wingers and especially forwards presented higher odds of running with the ball than central backs. This fact reveals how the technical and tactical demands of the game depend on the specific role of each position, which is crucial to decide the spatial arrangement and organization of players according to their specific skills. It is interesting to mention that according to our descriptive data, central backs performed a high quantity of actions without defensive pressure and with a high level of offensive support, whereas the other playing positions had more defensive pressure and less offensive support. Under these tactical circumstances, midfielders, wingers and forwards would require more individual actions to move away from the pressing opponents.

In the same line, Saward et al. [28] observed that playing positions in youth soccer affects the development of match skills through the seasons, so they observed important tactical differences between positions. For instance, central backs performed more tackles/blocks/interceptions, midfielders were more focused on passing, while wide midfielders and forwards performed more dribbles and attacking actions such as crosses and shots. Furthermore, previous studies have also reported different physical demands according to playing positions in youth soccer players [29,30], which demonstrates that highly specialized technical, tactical and physical demands exist in youth soccer players. These findings keep open the debate about the suitability and consequences of early specialization in youth ages, so it has been shown that the variability in the practice of soccer during youth ages seems to be the best way to develop soccer skills in the long term [31,32].

Other tactical dimensions that showed a significant effect to increase the runs and dribbles with the ball were receiving the ball facing the opposing goal and not having offensive support. In this sense, the fact of not having offensive support requires an individual solution based on carrying the ball or dribbling, while receiving the ball facing forward creates an opportunity to progress towards the opposing goal and increases the possibility to create a goal-scoring opportunity.

Regarding the tactical performance, U10 players had lower odds of achieving a positive outcome in their actions in comparison with U12 players regardless of other contextual and tactical dimensions. Related to this finding, Sevil Serrano, Práxedes, García-González., Moreno and del Villar [33] performed a study where the aim was to analyze the tactical behavior of soccer players in real-game situations across the different stages of development. This study revealed that U10 players had lower average decision-making and a lower percentage of successful executions for total actions and in passing and dribbling, compared to U12 players. In light of this lower performance, these authors proposed to change the competitive format of U10 teams by reducing the number of players (i.e., 5v5) and the spatial dimensions, in order to improve the player participation and the tactical performance. In relation to this, Serra-Olivares, Garcia-López and Goncalves [34] observed that U12 players had a better space occupation and dispersion on the pitch than U11 players, who tend to be more focused on the ball, leading to a high concentration of players around it.

Other tactical dimensions also presented a combined and significant effect on the tactical outcome such as the field zone, body shape, defensive pressure, offensive support, type of attack and type of action. In this way, tactical situations that include receiving the ball in the offensive half, facing forward, being under defensive pressure, being without offensive support, engaging in a counterattack and carrying the ball or dribbling decreased the odds of achieving a positive outcome. These findings highlight the necessity of understanding the tactical context when evaluating the technical and tactical performance in soccer players.

This study is not exempt of limitations. Despite our tactical evaluation included the analysis of multiple interdependent dimensions, the only focus was offensive and based on the behavior of the player with the ball, so that other important behaviors such as defensive actions or movements off the ball were not evaluated. Moreover, this study was based on observational methodology and this process includes the observation, interpretation and recording of events that occur during the game. This method may not entirely capture the complex and interactive nature of individual tactical actions during the game as some authors claimed [35,36,37]. Finally, our study did not collect the physical characteristics of players, which can compromise the generalization of the findings.

However, this investigation has relevant practical applications for practitioners in youth soccer. On one hand, this study found tactical differences between U10 and U12 age categories and playing positions, which can guide coaches and especially soccer methodology directors when designing long-term player development training plans according to the age category. On the other hand, our research revealed important interactive effects of multiple tactical dimensions on the individual offensive performance in youth soccer players, which can help coaches to design representative training exercises [38] that include and modulate constraints based on these indicators such as the body shape when receiving, defensive pressure or type of action, among others [39].

To conclude, our findings revealed that U10 players, in comparison with U12 players, presented higher odds of running and dribbling with the ball vs. passing the ball, as well as lower odds of achieving offensive success. In addition, our study determined that technical-tactical dimensions such as the players’ body shape when receiving the ball, the offensive support, defensive pressure, collective type of attack and type of technical action presented a significant and combined effect on the offensive success regardless of the age category, playing position and match status.

## Figures and Tables

**Table 1:** Operational definitions for the dimensions included in the study, based on the INDISOC tool [15].

Tactical Dimension	Categories
**1. Field zone:** Zone of the field where the player receives or recovers the ball.	(a) **Defensive half:** the action starts in the half field nearer the own goal. (b) **Offensive half:** the action starts in the half field nearer the opposing goal.
**2. Body shape:** Body orientation with respect to the opponent’s goal when receiving the ball.	(a) **Facing forward:** the player’s chest is facing the opposing goal. (b) **Facing sideways:** the player’s chest is facing the right or left line in relation to the opposing goal. (c) **Facing backwards:** the player’s back is facing the opposite side of the opposing goal.
**3. Offensive support:** Number of passing options that the ball carrier possesses at the moment of receiving the ball possession.	(a) **Offensive support:** the ball carrier has open passing lanes with 1 or more teammates. (b) **No offensive support:** the ball carrier has no open passing lanes with his/her teammates.
**4. Defensive pressure:** Distance between the ball carrier and the immediate pressing opponent player(s) during the first three seconds of the ball possession.	(a) **Defensive pressure:** one or several opponent players pressure the ball carrier within the first 3 s of the possession (the defender(s) are located within 1.5 m of the player). (b) **Non-defensive pressure:** any player pressures the ball carrier during the first 3 s of the possession.
**5. Type of attack:** Degree of offensive directness in the offensive process [22,23,24]	(a) **Positional attack:** (a) the possession starts by winning the ball in play or restarting the game, (b) the opposing team has the opportunity to minimize surprise, reorganize their system and be prepared defensively and (c) the circulation of the ball takes place more in width than in depth [23] and (d) the intention of the team is to disorder the opponent using either fast (short and quick passes), direct (one long pass from defense to attack) or combinative play (short and non-penetrative passes). (b) **Counterattack:** (a) the possession starts by winning the ball in play, (b) the progression towards the goal attempts to utilize a degree of imbalance from start to the end with high tempo [22] (c) the circulation of the ball takes place more in depth than in width and the intention of the team is to exploit the space left by the opponent when they were attacking and (d) the opposing team does not have the opportunity to minimize surprise, reorganize their system and be prepared defensively.
**6. Type of action:** Behavior of the ball carrier since he/she receives the ball until the culmination of the action.	(a) **Receiving and passing:** the ball carrier uses one or few contacts with the ball to culminate the technical-tactical action. (b) **Running with the ball:** the ball carrier runs with the ball performing multiple touches, directional changes, or/and dribbles prior to culminating the action.
**7. Tactical outcome:** Final performance of the action, considering the success when passing/shooting/dribbling.	(a) **Positive outcome:** it is considered a successful action when there is one of the next outcomes: pass completed/goal/foul received, corner or throw in achieved. (b) **Negative outcome:** it is considered a non-successful action when there is one of the next outcomes: pass intercepted/missed/shot off target/ball out of play/ball lost by tackle/turnover/foul committed/no control of the ball.

**Table 2:** Descriptive characteristics of the sample.

Tactical Dimensions	Total		Playing Positions (%)				Age Category (%)	
	*n*	(%)	Central Defender	Winger	Midfielder	Forward	U10	U12
**Field zone**								
Defensive half	681	(54.6)	89.9	61.5	48.8	16.3	58.8	50.4
Offensive half	566	(45.4)	10.1	38.5	51.2	83.7	41.2	49.6
**Body shape**								
Backwards	224	(18.0)	5.6	8.7	19.6	40.1	20.9	15.0
Sideways	242	(19.4)	13.2	18.7	23.8	20.2	15.2	23.7
Forward	781	(62.6)	81.2	72.6	56.7	39.7	63.9	61.4
**Offensive support**								
Non-offensive support	109	(8.7)	2.1	10.0	3.7	24.9	4.3	13.2
Offensive support	1138	(91.3)	97.9	90.0	96.3	75.1	95.7	86.8
**Defensive pressure**								
Defensive pressure	904	(72.5)	58.9	63.9	77.2	90.3	83.1	61.8
Non-defensive pressure	343	(27.5)	41.1	36.1	22.8	9.7	16.9	38.2
**Type of attack**								
Organized attack	1078	(86.4)	90.6	89.6	86.9	77.4	87.4	85.5
Counterattack	169	(13.6)	9.4	10.4	13.1	22.6	12.6	14.5
**Type of action**								
Receiving and passing	868	(69.6)	16.0	29.4	32.4	44.4	65.7	73.6
Running with the ball	379	(30.4)	84.0	70.6	67.6	55.6	34.3	26.4
**Tactical performance**								
Positive outcome	710	(56.9)	65.5	57.5	59.2	43.2	53.4	60.5
Negative outcome	537	(43.1)	34.5	42.5	40.8	56.8	46.6	39.5

**Table 3:** Random effects of team identity on the type of action and tactical performance in individual ball possessions.

Possession Type	Estimate	Std. Error	Z	Sig	95% CI
**Type of action**	0.42	0.70	0.592	0.554	0.002–1.142
**Tactical performance**	0.084	0.71	1.179	0.239	0.016–0.441

**Table 4:** Multilevel mixed linear model to predict the type of action.

Contextual and Tactical Dimensions	Running with the Ball vs. Receiving and Passing (Univariate Analysis)		Running with the Ball vs. Receiving and Passing (Multivariate Analysis)	
	*B*	OR (95% CI)	*B*	OR (95% CI) ^b^
**Age**				
U12 ^a^				
U10	0.375	1.455 (1.141–1.856) **	0.601	1.823 (1.333–2.493) ***
**Playing position**				
Central defender ^a^				
Winger	0.918	2.504 (1.649–3.804) ***	0.923	2.516 (1.625–3.894) ***
Midfielder	0.944	2.507 (1.755–3.764) ***	0.978	2.660 (1.757–4.027) ***
Forward	1.520	4.573 (3.037–6.886) ***	1.488	4.426 (2.647–7.401) ***
**Initial zone**				
Defensive half ^a^				
Offensive half	0.566	1.761 (1.378–2.252) ***	0.067	1.070 (0.795–1.439)
**Body shape**				
Backwards ^a^				
Sideways	−0.141	0.868 (0.577–1.306)	0.182	1.200 (0.775–1.857)
Forward	0.152	1.164 (0.840–1.612)	0.585	1.794 (1.226–2.625) **
**Offensive support**				
Non-offensive support ^a^				
Offensive support	−1.219	0.296 (0.198–0.443) ***	−0.807	0.446 (0.283–0.703) **
**Defensive pressure**				
Defensive pressure ^a^				
Non-defensive pressure	−0.549	0.577 (0.432–0.771) ***	−0.197	0.821 (0.593–1.137)
**Type of attack**				
Organized attack ^a^				
Counterattack	0.364	1.438 (1.023–2.022) *	0.017	1.017 (0.700–1.477)

B = regression coefficient; OR = Odds Ratio; CI = Confidence interval for odds ratio; * = *p* > 0.05 ** = *p* > 0.01 *** = *p* > 0.001; ^a^ Reference category; ^b^ Adjusted for contextual variables (match status and match half).

**Table 5:** Multilevel mixed linear model to predict the tactical performance.

Contextual and Tactical Dimensions	Positive Outcome vs. Negative Outcome (Univariate Analysis)		Positive Outcome vs. Negative Outcome (Multivariate Analysis)	
	*B*	OR (95% CI)	*B*	OR (95% CI) ^b^
**Age**				
U12 ^a^				
U10	−0.247	0.781 (0.543–1.123)	−0.359	0.698 (0.525–0.928) *
**Playing position**				
Central defender ^a^				
Winger	−0.429	0.651 (0.454–0.935) *	−0.163	0.850 (0.580–1.243)
Midfielder	−0.301	0.740 (0.536–1.022)	0.015	1.015 (0.703–1.466)
Forward	−0.912	0.402 (0.279–0.577) ***	−0.226	0.797 (0.495–1.286)
**Initial zone**				
Defensive half ^a^				
Offensive half	−0.773	0.462 (0.364–0.586) ***	0.509	0.601 (0.452–0.800) ***
**Body shape**				
Backwards ^a^				
Sideways	0.330	1.391 (0.941–2.056)	−0.447	1.292 (0.848–1.967)
Forward	−0.338	0.713 (0.524–0.971) *	0.256	0.640 (0.445–0.921) *
**Offensive support**				
Non-offensive support ^a^				
Offensive support	1.631	5.112 (3.202–8.160) ***	1.096	2.991 (1.789–5.000) ***
**Defensive pressure**				
Defensive pressure ^a^				
Non-defensive pressure	0.696	2.007 (1.523–2.644) ***	0.489	1.630 (1.204–2.207) **
**Type of attack**				
Organized attack ^a^				
Counterattack	−0.881	0.414 (0.293–0.586) ***	−0.597	0.551 (0.380–0.797) **
**Type of action**				
Receiving and passing ^a^				
Running with the ball	−0.900	0.407 (0.316–0.523) ***	−0.676	0.509 (0.388–0.667) ***

B = regression coefficient; OR = Odds Ratio; CI = Confidence interval for odds ratio; * = *p* > 0.05 ** = *p* > 0.01 *** = *p* > 0.001; ^a^ Reference category; ^b^ Adjusted for contextual variables (match status and match half).

## Data Availability

The data that support the findings of this study are available upon request to the corresponding author.
